# Supplementary material for: Emotional Eating, Health Behaviours, and Obesity in Children: A 12-Country Cross-Sectional Study
Source: Nutrients. 2019 Feb 7;11(2):351. doi: 10.3390/nu11020351 (PMC6412589; doi:10.3390/nu11020351)
Supplement: Supplementary file 1 [file nutrients-11-00351-s001.pdf]

Supplementary Materials: Emotional eating, health behaviours, and obesity in children: a 12-country cross-sectional study

Elli Jalo, Hanna Konttinen, Henna Vepsäläinen, Jean-Philippe Chaput, Gang Hu, Carol Maher, José Maia, Olga L. Sarmiento, Martyn Standage, Catrine Tudor-Locke, Peter T. Katzmarzyk, and Mikael Fogelholm

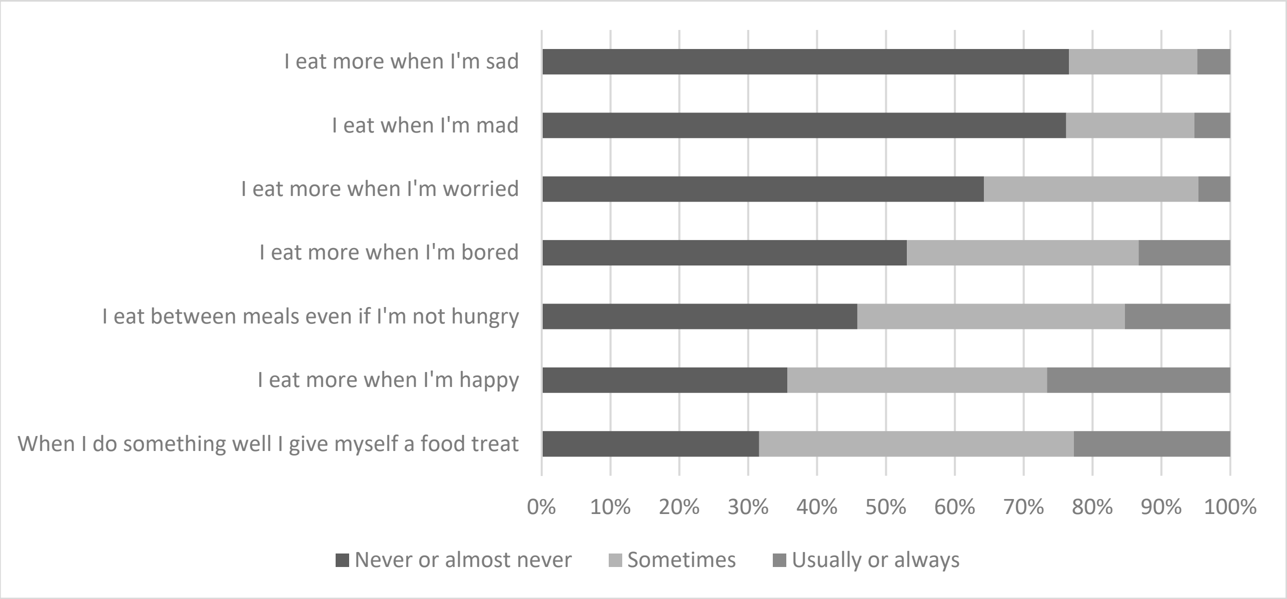

Figure S1. Item level frequencies of the original Emotion-Induced Eating Scale, n = 5426.
